# Unravelling staphylococcal small-colony variants in cardiac implantable electronic device infections: clinical characteristics, management, and genomic insights

**DOI:** 10.3389/fcimb.2023.1321626

**Published:** 2024-01-08

**Authors:** Si Liu, Hongbin Chen, Fangjie Xu, Fengning Chen, Yuyao Yin, Xiaoyang Zhang, Shangyu Tu, Hui Wang

**Affiliations:** ^1^ Department of Clinical Laboratory, Peking University People’s Hospital, Beijing, China; ^2^ Department of Clinical Laboratory, Urumqi Friendship Hospital, Urumqi, China

**Keywords:** small-colony variants (SCVs), *Staphylococcus epidermidis*, Cardiac implantable electronic device (CIED) infection, whole-genome sequencing (WGS), epidemiological survey

## Abstract

**Objectives:**

Staphylococcal small-colony variants (SCVs) are common in cardiac implantable electronic device (CIED) infections. This is the first retrospective and multi-case study on CIED infections due to staphylococcal SCVs, aiming to provide a theoretical basis for the clinical management of CIED and device-related infections caused by staphylococcal SCVs.

**Methods:**

Ninety patients with culture positive CIED infections were enrolled between 2021 and 2022. We compared the demographic and clinical characteristics of patients with and without SCVs and performed genomic studies on SCVs isolates.

**Results:**

Compared to patients without SCVs, those with SCVs had a longer primary pacemaker implantation time and were more likely to have a history of device replacement and infection. They showed upregulated inflammatory indicators, especially higher NEUT% (52.6 vs. 26.8%, *P* = 0.032) and they had longer hospital stays (median 13 vs. 12 days, *P* = 0.012). Comparative genomics analysis was performed on *Staphylococcus epidermidis* wild-type and SCVs. Some genes were identified, including *aap*, genes encoding adhesin, CHAP domain-containing protein, LPXTG cell wall anchor domain-containing protein, and YSIRK-type signal peptide-containing protein.

**Conclusion:**

Staphylococcal SCVs affect the clinical characteristics of CIED infections. The process of staphylococcal SCVs adherence, biofilm formation, and interaction with neutrophils play a vital role.

## 1 Introduction

Cardiac implantable electronic devices (CIED) such as cardiac pacemakers (CPs), implantable cardioverter defibrillators (ICDs), and devices for cardiac resynchronisation therapy (CRT) play vital roles in a variety of cardiac diseases (Döring et al., 2018). Unfortunately, infections after CIED implantation pose a significant public health concern (Tarakji et al., 2019).

Numerous studies have reported that staphylococci are the main culprit (Chua et al., 2000; Sohail et al., 2007a; Athan et al., 2012). Some infections are predominantly *Staphylococcus aureus* (Athan et al., 2012), while others are dominated by coagulase-negative staphylococci (CoNS) (Chua et al., 2000; Sohail et al., 2007a). Notably, staphylococcal small-colony variants (SCVs) have been reported in several cases (von Eiff et al., 1999; Seifert et al., 2003; Maduka-Ezeh et al., 2012; Tumbarello et al., 2012; Chen et al., 2018; Kussmann et al., 2018; Liu et al., 2023), both in *S. aureus* and CoNS. However, large-scale epidemiological surveys are lacking at the present stage.

Staphylococcal SCVs often emerge in recurrent and persistent infections such as device-associated infections, bone and tissue infections, and airway infections in patients with cystic fibrosis (Kahl et al., 2016). They are characterised by a slow growth rate, atypical colony morphology, and unusual biochemical features, which shows as a quasi-dormant and persistent phenotype (Pascoe et al., 2014; Conlon et al., 2016; Lee et al., 2020; Gunn et al., 2021; Peyrusson et al., 2022). Moreover, they have a strong ability to survive inside host cells without being killed and an increased resistance to antibiotics than their wild-type (WT) counterparts, making clinical treatment a challenge (Proctor et al., 2006).

However, although there are a number of studies on CIED staphylococcal SCVs infection, most are case reports, and there are no epidemiological investigations. To our knowledge, this is the first systematic, multi-case, retrospective analysis of CIED SCV infections. Molecular, epidemiological, and genomic analyses were performed on the isolates.

Herein, we pose several questions. What factors are most likely to contribute to the development of SCVs? How do SCVs affect patient clinical management and prognosis? Which features of these SCVs are superior to their WT counterparts isolated from the same patients, making them persistent and difficult to remove from the patients? We enrolled patients with CIED infection with or without SCVs for comparison of demographics and clinical characteristics, and collected the isolates for molecular epidemiology and genomic studies, aiming to provide a theoretical basis for clinical management of patients with CIED infection and clues for subsequent research on staphylococcal SCVs device-related infections.

## 2 Materials and methods

### 2.1 Study design, patients, and strains

This single-centre retrospective cohort study was conducted at the Peking University People’s Hospital. From January 2021 to January 2022, 90 patients with culture positive CIED infection were enrolled and case information was collected (**Supplementary Figure 1**). All strains from the patients were isolated when they were admitted to the hospital and stored at -80°C for further use. In this study, 149 isolates were collected and confirmed using MALDI-TOF (TOF/TOF) mass spectrometry (Autoflex Speed, Bruker, Germany). Antimicrobial susceptibility was evaluated according to the Clinical and Laboratory Standards Institute guidelines. The enrolled patients were grouped according to the presence or absence of the SCVs.

### 2.2 Culturing conditions

All isolates were plated on blood agar plate (Columbia, 5% sheep blood) and incubated overnight at 37°C. Single colony was selected from the plates and incubated overnight in tryptone soy broth (Oxoid, Thermo Scientific™) with shaking and used for DNA extraction. Alternatively, we incubated plates with both SCVs and WT for 24h in order to quantify colony size.

### 2.3 Statistical analysis

We compared two mutually exclusive groups of patients: (i) patients with staphylococcal SCVs and (ii) patients without staphylococcal SCVs. Quantitative variables were expressed as median with interquartile range. Qualitative variables were expressed as frequencies and percentages. Continuous variables were compared using the Mann–Whitney U test or Student’s t-test, as appropriate. Categorical variables were compared using the Pearson’s chi-square test, continuity correction, or Fisher’s exact test, as appropriate. *P* < 0.05 was considered statistically significant. SPSS Statistics (version 23.0, IBM Corp., Armonk, NY, USA) was used for all statistical analyses.

### 2.4 Library construction, quality control and whole-genome sequencing (WGS)

Total genomic DNA of the 39 isolates was extracted using a TIANamp Bacteria DNA Kit (Tiangen Biotech Co. Ltd., Beijing, China). A total amount of 0.2 μg DNA per sample was used as input material for the DNA library preparations. Sequencing library was generated using Rapid Plus DNA Lib Prep Kit for Illumina (Cat.No.RK20208) fllowing manufacturer’s recommendations and index codes were added to each sample. Briefly, genomic DNA sample was fragmented by sonication to a size of 350 bp. Then DNA fragments were endpolished, A-tailed, and ligated with the full-length adapter for Illumina sequencing, followed by further PCR amplification. After PCR products were purified by AMPure XP system (Beverly, USA). Subsequently, library quality was assessed on the Agilent 5400 system (Agilent, USA) and quantified by QPCR (1.5 nM). The qualified libraries were pooled and sequenced on Illumina platforms (Illumina Inc., San Diego, CA, USA).

### 2.5 Bioinformatic analysis

The Mash (Ondov et al., 2016) tool was used to identify the best-matching chromosomal reference. The reads were mapped and single nucleotide polymorphisms (SNPs) were identified using breseq (Deatherage and Barrick, 2014). Sequencing reads were assembled using SPAdes v3.13.0 (Bankevich et al., 2012), then performed quality assessment by QUAST v4.6.3 (Gurevich et al., 2013) (**Supplementary Report**). Contigs were annotated using Prokka v1.13.7 (Seemann, 2014). For phylogenetic analysis, the core genome of all strains was identified using the pangenome analysis pipeline Roary v3.12.2 (Page et al., 2015). Maximum likelihood phylogenetic trees were constructed using IQ-TREE software (Nguyen et al., 2015). Finally, a tree was plotted and annotated using the iTOL Web tool (https://itol.embl.de/). Multilocus sequence typing (MLST) was performed using the Center for Genomic Epidemiology tools (www.genomicepidemiology.org/services/). Resistance genes were identified using ResFinder (https://cge.food.dtu.dk/services/ResFinder/) (Florensa et al., 2022) and the Comprehensive Antibiotic Resistance Database (CARD; https://card.mcmaster.ca) (Alcock et al., 2023), and virulence genes were identified using VirulenceFinder (cge. food. dtu. dk/services/VirulenceFinder/) and the Virulence Factor Database (www.mgc.ac.cn/VFs/main.htm) (Liu et al., 2022).

## 3 Results

### 3.1 Demographic and clinical characteristics

Ninety patients with culture positive CIED infection were included in the cohort (**Supplementary Figure 1**). Of these, 19 (21.1%) had isolated staphylococcal SCVs and 71 (78.9%) were without SCVs. We enumerated the relevant features of all patients and compared the two groups with and without SCV, as shown in **Table 1**.

**Table 1:** Demographic and clinical characteristics.

	Total (n=90)	Patients without SCVs (n=71)	Patients with SCVs (n=19)	*P* Value
Demographic features				
Sex, male	61 (67.8%)	47 (66.2%)	14 (73.7%)	0.535
Age, years	68 (59–74)	68 (61–74)	61(58–74)	0.54
Implant-related feature				
CIED type				
CP	74 (82.2%)	57 (80.3%)	17 (89.5%)	0.553
ICD	6 (6.7%)	6 (8.5%)	0	0.427
CRT	10 (11.1%)	8 (11.3%)	2 (10.5%)	1
Interval between primary pacemaker implantation and the current infection, years	9 (3–12.25)	8 (2.5–12)	10 (6–14)	0.143
Times the device replacement/revision before the current infection, times				
0	44 (48.9%)	38 (53.5%)	6 (31.6%)	0.089
1	35 (38.9%)	24 (33.8%)	11 (57.9%)	0.056
2	11 (12.2%)	9 (12.7%)	2 (10.5%)	1
Interval between the last device replacement/revision and the current infection, years	2 (1–3)	2 (1–3)	3 (1–4)	0.425
History of device infection	15 (16.7%)	9 (12.7%)	6 (31.6%)	0.106
Implantation site inflammatory reaction	80 (88.9%)	61 (85.9%)	19 (100%)	0.185
Systemic inflammatory reaction	11 (12.2%)	6 (8.5%)	5 (26.3%)	0.086
Fever > 38**°**C	19 (21.1%)	14 (19.7%)	5 (26.3%)	0.757
Infection degree				
Superficial infection	5 (5.6%)	5 (7%)	0	0.531
Pocket infection	75 (83.3%)	59 (83.1%)	16 (84.2%)	1
Bacteremia	5 (5.6%)	5 (7%)	0	0.531
Infective endocarditis	5 (5.6%)	2 (2.8%)	3 (15.8%)	0.103
Underlying diseases				
Diabetes mellitus	33 (36.7%)	24 (33.8%)	9 (47.4%)	0.276
Renal insufficiency	8 (8.9%)	7 (9.9%)	1 (5.3%)	0.864
COPD	2 (2.2%)	1 (1.4%)	1 (5.3%)	0.38
Cancer	4 (4.4%)	3 (4.2%)	1 (5.3%)	1
Anticoagulants	3 (3.3%)	2 (2.8%)	1 (5.3%)	0.513
Skin disease	2 (2.2%)	1 (1.4%)	1 (5.3%)	0.38
Thyroid disorder	6 (6.7%)	5 (7%)	1 (5.3%)	1
Cerebral infarction	8 (8.9%)	7 (9.9%)	1 (5.3%)	0.864
Smoking	13 (14.4%)	11 (15.5%)	2 (10.5%)	0.857
HLP	15 (16.7%)	12 (16.9%)	3 (15.8%)	1
Hypertension	39 (43.3%)	31 (43.7%)	8 (42.1%)	0.903
CHD	14 (15.6%)	12 (16.9%)	2 (10.5%)	0.745
Heart failure/DCM	13 (14.4%)	11 (15.5%)	2 (10.5%)	0.857
Atrial fibrillation	8 (8.9%)	7 (9.9%)	1 (5.3%)	0.864
Laboratory examination				
Higher WBC count	17 (18.9%)	12 (16.9%)	5 (26.3%)	0.548
Higher NEUT%	29 (32.2%)	19 (26.8%)	10 (52.6%)	0.032
Anaemia	46 (51.1%)	34 (47.9%)	12 (63.2%)	0.454
CRP, mg/L	10.35 (1.60–33.78)	6.55 (1.25–27.93)	28.05 (7.73–73.63)	0.057
PCT, ng/ml	0.05 (0.03–0.15)	0.05 (0.03–0.083)	0.12 (0.03–1.56)	0.074
ESR, mm/h	28 (7–38)	17 (6.5–35)	35.5 (25–50.25)	0.069
BNP, pg/ml	127 (53–250)	123.5 (53–251)	154 (42–290.5)	0.844
Abnormal myocardial injury biomarkers (Mb, hs-TnI, CK-MB)	39 (43.3%)	30 (42.3%)	9 (47.4%)	0.648
Echocardiography				
Vegetation on device lead	6 (6.7%)	3 (4.2%)	3 (15.8%)	0.202
Any valvular vegetation	2 (2.2%)	1 (1.4%)	1 (5.3%)	0.38
Clinical treatment and outcomes				
Temporary pacing	42 (46.7%)	32 (45.1%)	10 (52.6%)	0.557
Reimplantation	69 (76.7%)	54 (76.1%)	15 (78.9%)	1
Interval between extraction and reimplantation, days	2 (1-6)	2.5 (1-6)	1 (1-6)	0.492
Length of stay, days	12 (10-15.25)	12 (9-14)	13 (12-20)	0.012
Antibiotics adjustment	31 (34.4%)	22 (31%)	9 (47.4%)	0.182
Death	2 (2.2%)	2 (2.8%)	0	1
Microbiological features				
*Staphylococcus aureus*	11 (12.2%)	10 (14.1%)	1 (5.3%)	0.517
*Staphylococcus epidermidis*	55 (61.1%)	40 (56.3%)	15 (78.9%)	0.073
*Staphylococcus hominis*	22 (24.4%)	15 (21.1%)	7 (36.8%)	0.265
*Staphylococcus haemolyticus*	7 (7.8%)	6 (8.5%)	1 (5.3%)	1
*Staphylococcus capitis*	7 (7.8%)	5 (7%)	2 (10.5%)	0.983
*Staphylococcus caprae*	2 (2.2%)	2 (2.8%)	0	1
*Staphylococcus warneri*	2 (2.2%)	1 (1.4%)	1 (5.3%)	0.38
*Staphylococcus cohnii*	1 (1.1%)	1 (1.4%)	0	1
*Staphylococcus pettenkoferi*	1 (1.1%)	0	1 (5.3%)	0.211
*Staphylococcus lugdunensis*	1 (1.1%)	1 (1.4%)	0	1
Other species	7 (7.8%)	6 (8.5%)	1 (5.3%)	1

Quantitative variables are expressed as median (interquartile range), and qualitative variables are expressed as numbers (%).

SCVs, small-colony variants; CIED, Cardiac implantable electronic devices; CP, cardiac pacemaker; ICD, implantable cardioverter defibrillator; CRT, cardiac resynchronization therapy; COPD, chronic obstructive pulmonary disease; HLP, hyperlipidaemia; CHD, coronary heart disease; DCM, dilated cardiomyopathy; WBC, white blood cell; NEU%, neutrophil ratio; CRP, C-reactive protein; PCT, procalcitonin; ESR, erythrocyte sedimentation rate; BNP, brain natriuretic peptide.

### 3.2 Isolates profiles and antimicrobial susceptibility testing

A total of 142 *Staphylococcus* strains and seven other species were isolated from the pacemaker, pacemaker pocket, or lead specimens of 90 patients. *S. epidermidis* accounted for more than half of the total number of isolates (55.7%), followed by *Staphylococcus hominis* and *S. aureus* (16.1 and 8.1%, respectively) (**Supplementary Figure 2**).

Clinical antimicrobial susceptibility testing of 142 *Staphylococcus* isolates was performed and duplicate results from one patient’s specimen were removed. Methicillin-resistant *Staphylococcus* accounted for 53.2% of the total, methicillin-resistant coagulase-negative staphylococci, and methicillin-resistant *S. aureus* occupied 55.1, and 33.3% in CoNS and *S. aureus*, respectively. All *Staphylococcus* isolates were susceptible to vancomycin, daptomycin, linezolid, and tigecycline, while 2.2% were intermediate to teicoplanin. All *S. aureus* strains were susceptible to ceftaroline (**Supplementary Table 1**). CoNS WT strains showed more resistance to most antimicrobials, especially erythromycin, with resistance rates of 73.8 and 34.8% in CoNS WT and CoNS SCVs, respectively. However, rifampin showed 8.7% resistance in CoNS SCVs and only 4.7% resistance in the CoNS WT (**Supplementary Table 2**).

### 3.3 Molecular epidemiology of staphylococcal SCVs

**Figure 1:**
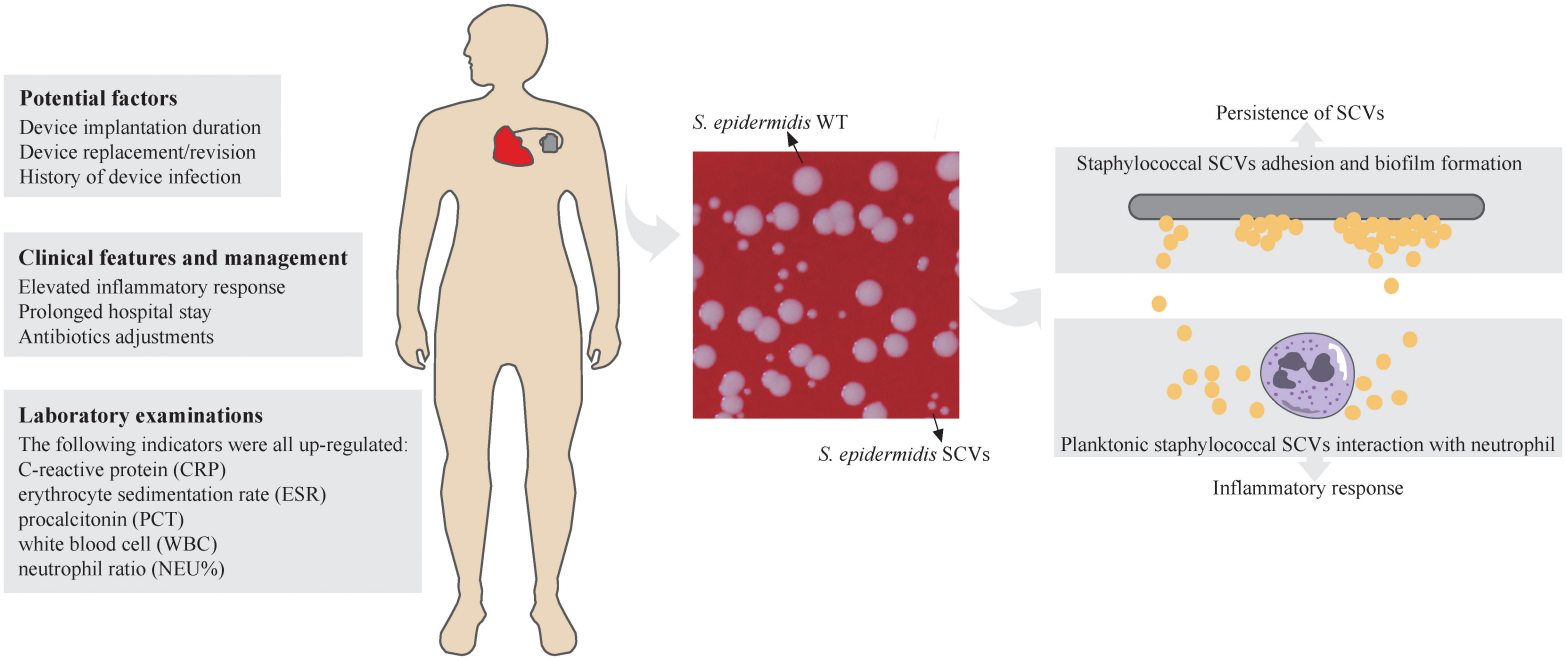
Schematic representation of Staphylococcal SCVs in CIED infection. The potential factors, clinical features and management, and laboratory examinations of CIED infection of staphylococcal SCVs were listed. Colony morphology of *S. epidermidis* WT and SCVs was showed on blood agar plate (Columbia, 5% sheep blood) at 37°C overnight. The diameters of thirty colonies (only ten were shown) were measured by imageJ and analysed by Student’s t-test. Adhesion and biofilm formation of staphylococcal SCVs on the device contribute to persistence of SCVs and are difficult to clear. The interaction between planktonic staphylococcal SCVs and neutrophils leads to inflammatory response in the host. ****, *P* ≤ 0.0001.

**Figure 2:**
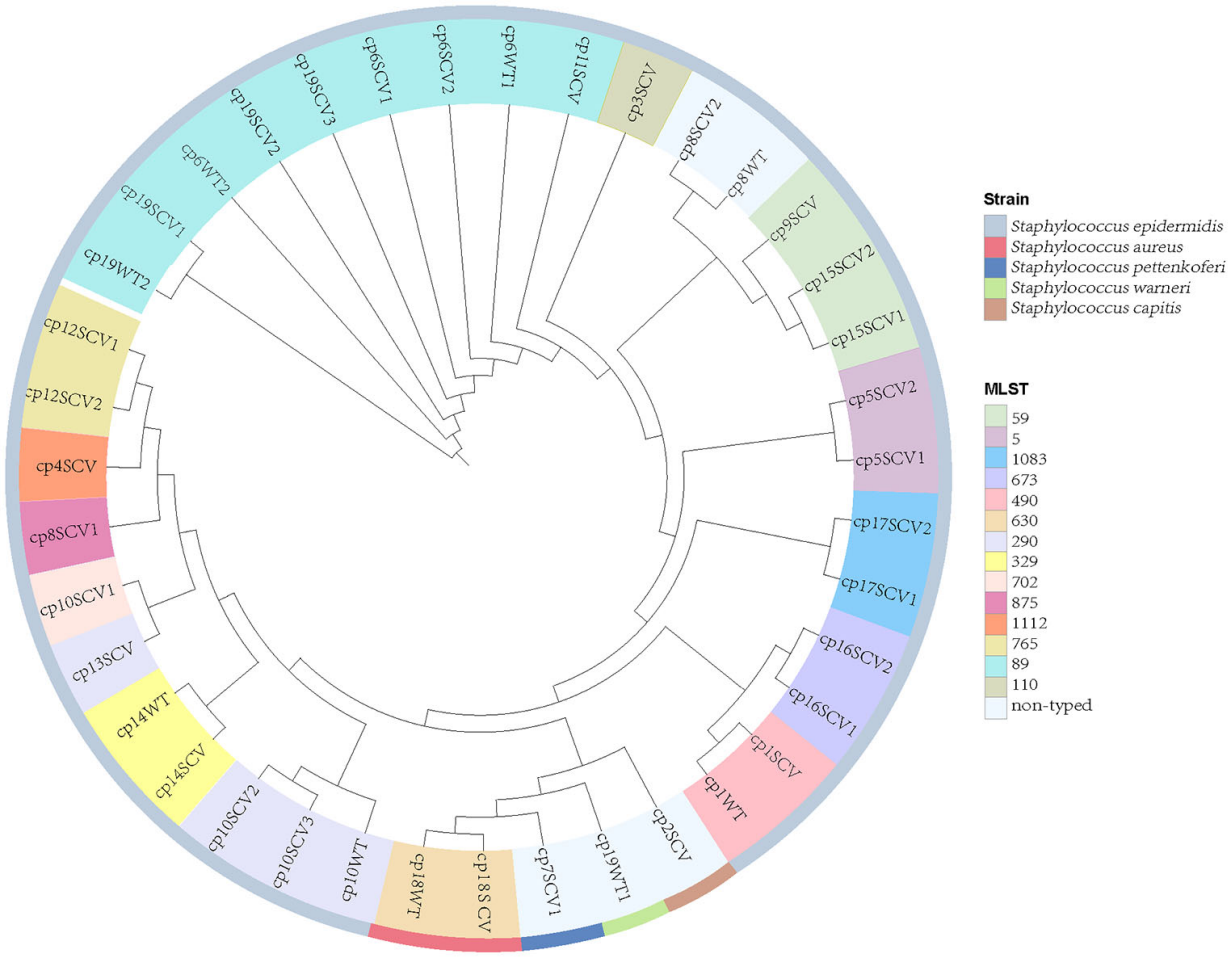
Phylogeny and multilocus sequence typing (MLST) of 39 staphylococcal SCVs and WT strains. Nine WT strains isolated from the same specimen with SCVs. Each branch in the tree represented one isolate. Information on MLST and strain species are mapped on the tree from inner to outer circle.

Thirty staphylococcal SCVs were isolated from 19/90 patients (cp1–cp19), meanwhile, nine WT strains were isolated from the same specimen. The WT and SCV colony morphologies of one pair of these *S. epidermidis* were shown in **Figure 1**, with significant differences in size. The 39 isolates consist of five species, including *S. epidermidis* (34/39), *S. aureus* (2/39), *Staphylococcus pettenkoferi* (1/39), *Staphylococcus warneri* (1/39), and *Staphylococcus capitis* (1/39). WGS was performed on these strains. A phylogenetic tree was constructed on *S. epidermidis* isolates (**Figure 2**). Comparable strain pairs with close genetic relationships were selected for subsequent studies. Multilocus sequence typing (MLST) of these strains was scattered and largely patient-related, with three patients isolating ST89 strains (**Figure 2**).

**Figure 3:**
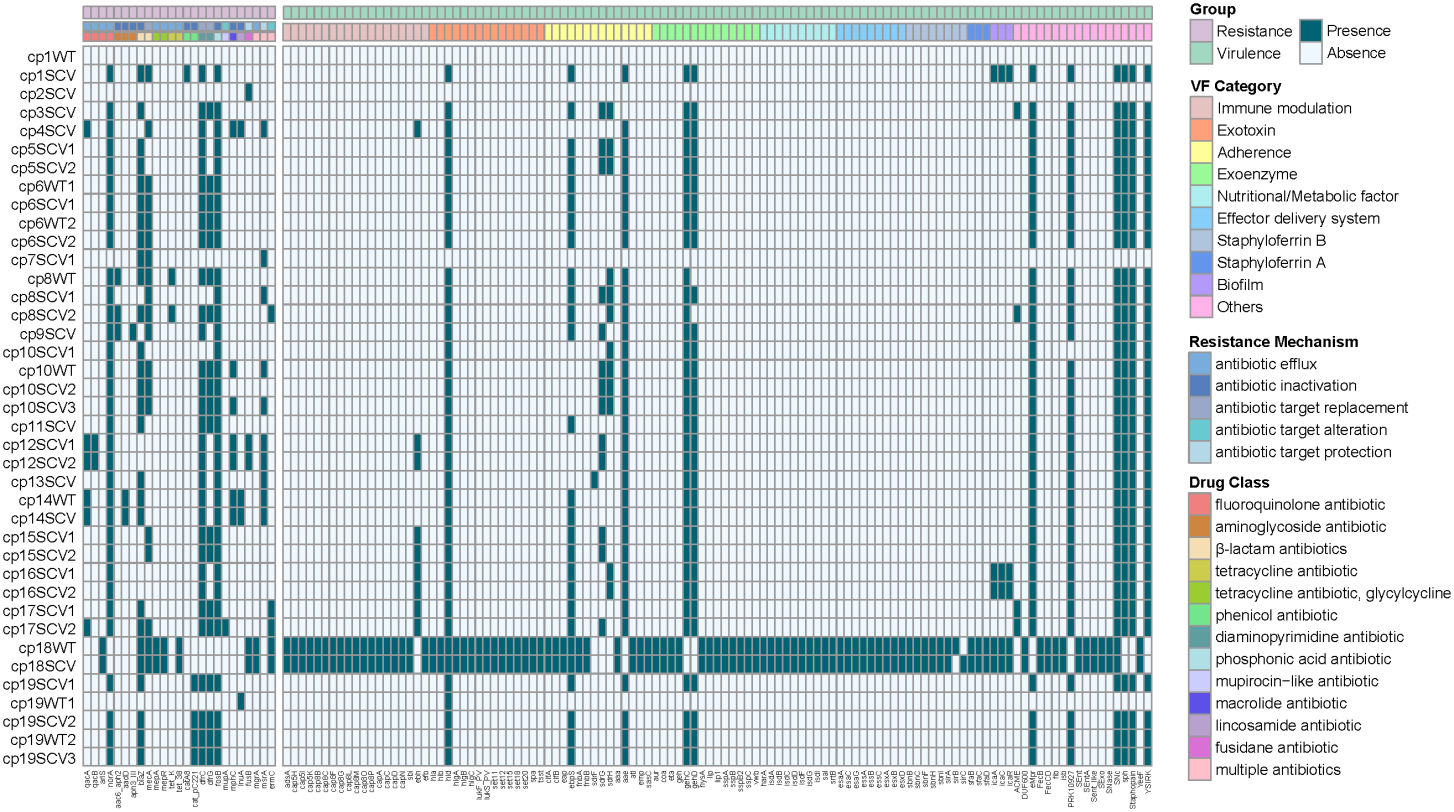
Heatmap of antibiotic resistance and virulence genes among the 39 staphylococcal SCVs and WT isolates. The “virulence factor (VF) Category” of virulence genes was classified based on Virulence Factor Database (VFDB) website (www.mgc.ac.cn/VFs/main.htm). The “Resistance Mechanism” and “Drug Class” of resistance genes were categorized by Comprehensive Antibiotic Resistance Database (CARD) website (//card.mcmaster.ca/).

Among 39 isolates, 25 antibiotic resistance genes and 113 virulence genes were identified (**Figure 3**). Genes resistant to fluoroquinolone antibiotic (*norA*), β-lactam antibiotics (*blaZ*, *mecA*), diaminopyrimidine antibiotic (*dfrC*, *dfrG*), and phosphonic acid antibiotic (*fosB*) were detected in half or more strains, mainly through antibiotic efflux, inactivation or target replacement to resistance. Most virulence genes were discovered in *S. aureus* (cp18WT and cp18SCV), while some were frequently detected in CoNS, such as *hld*, *ebpS*, *sdrG*, *sdrH*, *aae*, *gehC*, and *gehD*, etc. By categorising virulence factors, we found that those related to adhesion were the most widely distributed, including *ebpS*, *sdrG*, *sdrH*, and *aae*. However, there were no differences in the resistance and virulence genes between the SCVs and WT strains isolated from the same patient.

### 3.4 SNPs related to staphylococcal SCVs

Owing to the limited information available on resistance and virulence genes alone, we aimed to identify SNPs between staphylococcal WT and SCVs based on WGS to identify key genes related to SCVs formation and persistence. We selected *S. epidermidis* WT and SCVs that were isolated from one patient, and then selected strains close to the phylogenetic tree (**Figure 2**). Subsequently, eight pairs of *S. epidermidis* WT and SCVs were obtained from six patients (cp1, cp6, cp8, cp10, cp14, and cp19) to explore the universal and vital factors associated with SCVs. Numerous SNPs were detected in the CDS of genes and intergenic regions of the six patients (**Supplementary Figure 3**; **Supplementary Table**). After filtering, the 17 mutated genes shared by two or more patients are listed in **Table 2** and were divided into classes based on the UniProt website and Gene Ontology database.

**Table 2:** Mutated genes shared by two or more patients.

Class	Gene name/ID	Protein	Strains
**Cell wall**	*aap*	accumulation‐associated protein	cp1WT/cp1SCV cp8WT/cp8SCV2
	*EQW00_01465*	adhesin	cp1WT/cp1SCV cp8WT/cp8SCV2
	*EQW00_02070*	CHAP domain‐containing protein	cp1WT/cp1SCV cp10WT/cp10SCV2 cp10WT/cp10SCV3
	*EQW00_00920*	LPXTG cell wall anchor domain‐containing protein	cp1WT/cp1SCV cp8WT/cp8SCV2
	*EQW00_08490*	YSIRK‐type signal peptide‐containing protein	cp1WT/cp1SCV cp10WT/cp10SCV2 cp10WT/cp10SCV3
	*EQW00_10990*	YSIRK‐type signal peptide‐containing protein	cp1WT/cp1SCV cp8WT/cp8SCV2 cp10WT/cp10SCV2 cp10WT/cp10SCV3
**DNA-binding**	*EQW00_00200*	DNA primase	cp1WT/cp1SCV cp8WT/cp8SCV2
	*EQW00_00250*	SAM‐dependent DNA methyltransferase	cp1WT/cp1SCV cp8WT/cp8SCV2
**Membrane**	*EQW00_07660*	ABC transporter permease	cp8WT/cp8SCV2 cp10WT/cp10SCV2 cp10WT/cp10SCV3
	*EQW00_00780*	tandem‐type lipoprotein	cp8WT/cp8SCV2 cp10WT/cp10SCV2 cp10WT/cp10SCV3
**Other**	*EQW00_09465*	IS1182-like element ISSep1 family transposase	cp8WT/cp8SCV2 cp10WT/cp10SCV2 cp10WT/cp10SCV3
	*EQW00_00140*	IS6 family transposase	cp6WT1/cp6SCV1 cp6WT1/cp6SCV2 cp8WT/cp8SCV2
	*EQW00_01285*	2‐oxo acid dehydrogenase subunit E2	cp1WT/cp1SCV cp10WT/cp10SCV3
	*EQW00_00380*	DUF1643 domain‐containing protein	cp1WT/cp1SCV cp8WT/cp8SCV2
	*EQW00_00285*	DUF1643 domain‐containing protein	cp8WT/cp8SCV2 cp10WT/cp10SCV2 cp10WT/cp10SCV3
	*ebh*	hyperosmolarity resistance protein Ebh	cp1WT/cp1SCV cp10WT/cp10SCV2 cp10WT/cp10SCV3
	*EQW00_07700*	phage head morphogenesis protein	cp1WT/cp1SCV cp8WT/cp8SCV2

The table shows only the genes with mutations located in the CDS. The ‘Class’ is manually annotated based on the Uniprot website (www.uniprot.org). The ‘Gene’ and ‘Protein’ are extracted from *S. epidermidis* strain ATCC 14990 chromosome genome (GenBank: CP035288.1). In the ‘Strains’, each row is two comparable *S. epidermidis* strains (wild-type and SCVs) of the same patient which are closely related on the phylogenetic tree.

Approximately one-third of these genes were associated with the cell wall. Accumulation-associated protein (Aap) contributes to *S. epidermidis* corneocyte adherence (Roy et al., 2021) and biofilms formation on abiotic surfaces (Schaeffer et al., 2015). In addition, surface adhesins enable it to attach to the host and form biofilms on implanted foreign bodies (Sabate Bresco et al., 2017), causing *S. epidermidis* to emerge as an important opportunistic pathogen in patients receiving medical devices. The CHAP domain is found in a wide range of protein architectures associated with several families of amidases, suggesting that many of these proteins have multiple peptidoglycan hydrolytic activities (Bateman and Rawlings, 2003). LPxTG cell wall anchor domain-containing proteins are known to be anchored to bacterial peptidoglycans by sortases involved in LPxTG protein-dependent biofilm formation (Khodaparast et al., 2016). YSIRK/GS motif signal peptides are involved in cell division in staphylococci through the delivery of surface proteins to unique locations in the cell wall envelope (DeDent et al., 2008).

## 4 Discussion

### 4.1 Potential factors for CIED infection caused by staphylococcal SCVs

Several studies have reported the underlying diseases in patients with CIED infections, such as diabetes mellitus (Bloom et al., 2006), renal dysfunction (Bloom et al., 2006; Lekkerkerker et al., 2009), COPD (Sohail et al., 2011), oral anticoagulants (Bloom et al., 2006; Lekkerkerker et al., 2009), long-term corticosteroid use (Sohail et al., 2007b; Cengiz et al., 2010) and heart disease (Bloom et al., 2006; Hercé et al., 2013). Most of these factors were considered in our study and some additional factors have been included (**Table 1**). However, we did not find any underlying diseases related to SCVs in these patients. Also, age and sex have no correlation to SCVs, which were reported in CIED infections (Bloom et al., 2006; Cengiz et al., 2010; Johansen et al., 2011).

Device replacement and a history of device infection have been reported as contributors to CIED infections in numerous studies (Bloom et al., 2006; Klug et al., 2007; Sohail et al., 2007b; Lekkerkerker et al., 2009; Landolina et al., 2011). Although there was no statistically significant difference, patients with isolated SCVs were more likely to have a history of device replacement or revision and previous device infection based on this cohort, which was regarded as a potential factor contributing to the development of SCVs. SCVs often cause persistent and recurrent infections. Coincidentally, we found that the duration of primary pacemaker implantation was longer and the risk of SCVs formation was higher, although the difference was not statistically significant (**Table 1**).

### 4.2 Inflammatory response in patients with staphylococcal SCVs

von Eiff et al. reported two cases of pacemaker electrode infections caused by SCVs in *S. epidermidis* and *S. capitis* (von Eiff et al., 1999). All patients showed anaemia, elevated CRP protein, and ESR levels, and one had an increased white blood cell (WBC) count. Seifert et al. described a case of pacemaker-related bloodstream infection caused by *S. aureus* SCVs (Seifert et al., 2003). Laboratory studies also showed high CRP and increased ESR levels. Consistent with these reports, an elevated inflammatory response was observed in this study. Compared to patients without SCVs, patients with isolated SCVs had heightened CRP, ESR, and PCT levels and were more likely to have a higher WBC count and NEUT%, and the proportion of patients with anaemia was more than half. In addition to laboratory examinations, clinical manifestations found that systemic inflammatory reactions were more common in patients with SCVs.

When infection occurs, vascular neutrophils and phagocytic cells are actively recruited via chemotaxis to the infection site via chemokine gradients and pathogen-associated molecular patterns (Howden et al., 2023). The interactions between neutrophils and SCVs are complex. *S. aureus* SCVs infections increase neutrophilic inflammation (Bollar et al., 2022). In another study, *S. aureus* SCVs significantly reduced neutrophil chemotaxis relative to their WT counterparts (Guérillot et al., 2019). Although neutrophils have long been regarded as essential for host defence against *S. aureus* infection, they sometimes, facilitate *S. aureus* infection (Siwczak et al., 2022). By surviving inside neutrophils, they are used as nests for systemic dissemination to other organs (Siwczak et al., 2022; Howden et al., 2023). In addition to *S. aureus*, CoNS have also developed strategies to evade bactericidal attack by neutrophils (Cheung et al., 2010; Bogut and Magryś, 2021). *S. epidermidis* SCVs can survive inside macrophages and neutrophils for at least 3 days (Bogut and Magryś, 2021).

Thus, is a higher neutrophil proportion advantageous for controlling infection or making things worse? The mechanism underlying increased inflammatory response in patients with CIED infections caused by staphylococcal SCVs remains unknown.

### 4.3 Prolonged hospital stay and antibiotic adjustments in patients with staphylococcal SCVs

The formation of staphylococcal SCVs seemed to have no effect on temporary pacing and reimplantation operations; however, the length of hospital stay was longer in patients with SCVs than in those without. The median hospital stay was 8 days for patients with infections involving implantable cardiac electrophysiological devices (Chua et al., 2000). Prolonged hospital stay may have been caused by poor response to a variety of antimicrobials and vegetation formed on the device (von Eiff et al., 1999; Maduka-Ezeh et al., 2012; Tumbarello et al., 2012).

Preoperative administration of antibiotics is an effective way to reduce the risk of CIED infections (Tarakji et al., 2019). Beginning with the empiric antibiotic therapy, nearly half of the patients with SCVs infections in our study underwent antibiotic adjustments. It has been reported that the application of high-dose daptomycin in staphylococcal CIED endocarditis, may be associated with high microbiological responses and clinical success (Durante-Mangoni et al., 2012). Tumbarello et al. used a similar regimen to treat device-related endocarditis caused by staphylococcal SCVs (Tumbarello et al., 2012). In a case reported by Seifert et al., a patient with pacemaker lead infection caused by *S. aureus* SCVs underwent multiple antibiotic adjustments, including gentamicin, cefuroxime, meropenem, vancomycin, rifampin, and flucloxacillin (Seifert et al., 2003), indicating that antibiotic therapy for CIED infections caused by staphylococcal SCVs is frequently unsatisfactory.

According to the *in vitro* antimicrobial susceptibility testing in our study, compared to CoNS WT, CoNS SCVs showed similar resistance rates to most antibiotics, except that they were more susceptible to erythromycin and more resistant to rifampicin. However, *in vivo* antibiotic sensitivity may be more complex, posing challenges for clinical antibiotic adjustments.

### 4.4 Capabilities of adhesion and biofilm formation of *S. epidermidis* SCVs

Owing to the prolonged hospital stay of patients with CIED infections caused by staphylococcal SCVs, we aimed to determine the advantages of staphylococcal SCVs in terms of persistent survival inside the host. Numerous studies have reported interactions between *S. aureus* and its host as one of the foremost opportunistic bacterial pathogens in humans (Howden et al., 2023). Although CoNS is less aggressive than its close relative, *S. aureus*, they have the ability to evade host defences, and the biofilm mode of growth is believed to be a protective strategy (Schilcher and Horswill, 2020). First, we performed phylogenetic analysis and MLST of 39 staphylococcal WT and SCVs, and a few features were found (**Figure 2**). Subsequently, we identified and classified the virulence and resistance genes of the isolates and found that virulence factors related to adhesion had the most concentrated distribution (**Figure 3**). However, there were no significant differences in the expression of these genes between the WT and SCVs pairs isolated from the same patient. This may be because a part of the WT was on the way to becoming an SCVs.

In our cohort, *S. epidermidis* was responsible for more than half of the infections in both SCV and non-SCV groups. Therefore, we focused on *S. epidermidis* and mutant strains. To further explore the differences between *S. epidermidis* WT and SCVs, the whole genomes of the filtered isolates were analysed to identify significant SNPs. Many mutated genes were related to the cell wall (**Table 2**).

As expected, some mutations were strongly associated with adhesion and biofilm formation, such as accumulation-associated protein (*aap*) (Schaeffer et al., 2015; Roy et al., 2021), adhesin (*EQW00_01465*) (Sabate Bresco et al., 2017) and LPXTG cell wall anchor domain-containing protein (*EQW00_00920*) (Gill et al., 2005; Khodaparast et al., 2016), contributing by adhering to the implanted device or valvular tissue, and forming biofilm. The CHAP domain is associated with other domains that cleave peptidoglycan (Bateman and Rawlings, 2003). Peptidoglycan is an essential component of the cell wall that provides bacteria with a strong protective outer layer. Thus, CHAP domain-containing proteins play a vital role in cell wall biogenesis and degradation and are closely related to cell division. Another mutation, in the YSIRK-type signal peptide-containing protein, is also involved in cell division (DeDent et al., 2008) and was found in three patients (cp1, cp8, and cp10). In addition, DNA primase are essential for DNA replication (Larson et al., 2010). A mutation in DNA primase was also found in another report of *S. aureus* SCVs cardiac device-related endocarditis (Kussmann et al., 2018).

Our study is the first to provide a retrospective and multi-case research on staphylococcal SCVs CIED infections. We systematically analysed the demographics, clinical characteristics, and microbiological features of 90 patients grouped according to staphylococcal SCVs presence or absence, and performed genomic studies on SCV isolates. Based on this study, potential factors for SCVs formation in patients with CIED infection were identified. The clinical features and management were also analysed. Through comparative genomics analysis of *S. epidermidis* SCVs and WT isolates, key genes and physiological processes related to the formation and persistence of SCVs were identified (**Figure 1**). These findings provide a solid preliminary basis for the clinical characteristics and management of patients with CIED infections caused by staphylococcal SCVs, especially *S. epidermidis*, and subsequent research on staphylococcal SCVs device-related infections.

However, this study had some limitations. First, the data were collected retrospectively and the analysis was limited by the small number of patients. Second, only analyses at the genomic level were performed using bioinformatics without experimental verification, and the analyses mainly focus on *S. epidermidis* and was not representative of the whole. Third, the interaction between staphylococcal SCVs and the host can lead to inflammation; however, the underlying mechanism requires further studies.

In summary, our findings provide new insights into CIED infections caused by staphylococcal SCVs.

## Data availability statement

The datasets presented in this study can be found in online repositories. The names of the repository/repositories and accession number(s) can be found below: NCBI under BioProject accession number PRJNA1034826.

## Ethics statement

The studies involving humans were approved by Ethics Committee of Peking University People’s Hospital. The studies were conducted in accordance with the local legislation and institutional requirements. Written informed consent for participation was not required from the participants or the participants’ legal guardians/next of kin in accordance with the national legislation and institutional requirements. This study was conducted in accordance with the Declaration of Helsinki and was approved by the Ethics Committee of Peking University People’s Hospital (2022PHB073).

## Author contributions

SL: Conceptualization, Investigation, Visualization, Writing – original draft. HC: Funding acquisition, Methodology, Supervision, Writing – review & editing. FX: Data curation, Writing – original draft. FC: Formal analysis, Software, Writing – original draft. YY: Methodology, Writing – original draft. XZ: Validation, Writing – original draft. ST: Formal analysis, Writing – original draft. HW: Supervision, Writing – review & editing.
