# Supplementary material for: Unravelling staphylococcal small-colony variants in cardiac implantable electronic device infections: clinical characteristics, management, and genomic insights
Source: Front Cell Infect Microbiol. 2024 Jan 8;13:1321626. doi: 10.3389/fcimb.2023.1321626 (PMC10800868; doi:10.3389/fcimb.2023.1321626)
Supplement: Supplementary file 1 [file DataSheet_1.docx]

Supplementary Material

# Supplementary Data

Supplementary Table has been uploaded separately.

# Supplementary Figures and Tables

## Supplementary Figures


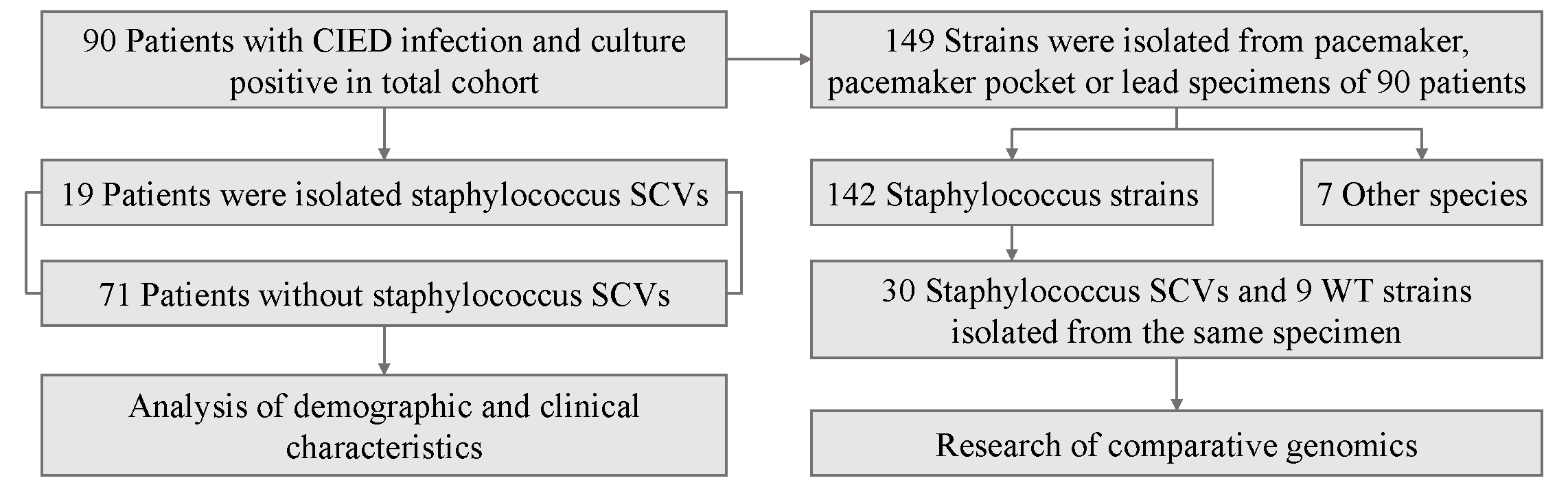


**Supplementary Figure 1.** Flow diagram of patient enrollment. Inclusion criteria as follows: (1) Patients admitted to department of cardiology and were diagnosed with CIED infections. (2) During the patient's hospital stay, cultures of pacemaker, pacemaker pocket or lead specimens were positive at least once. (3) These isolates can be obtained retrospectively. Totally, 90 patients were enrolled and 149 isolates were collected. Abbreviations: CIED, cardiac implantable electronic device; SCVs, small- colony variants; WT, wild-type.


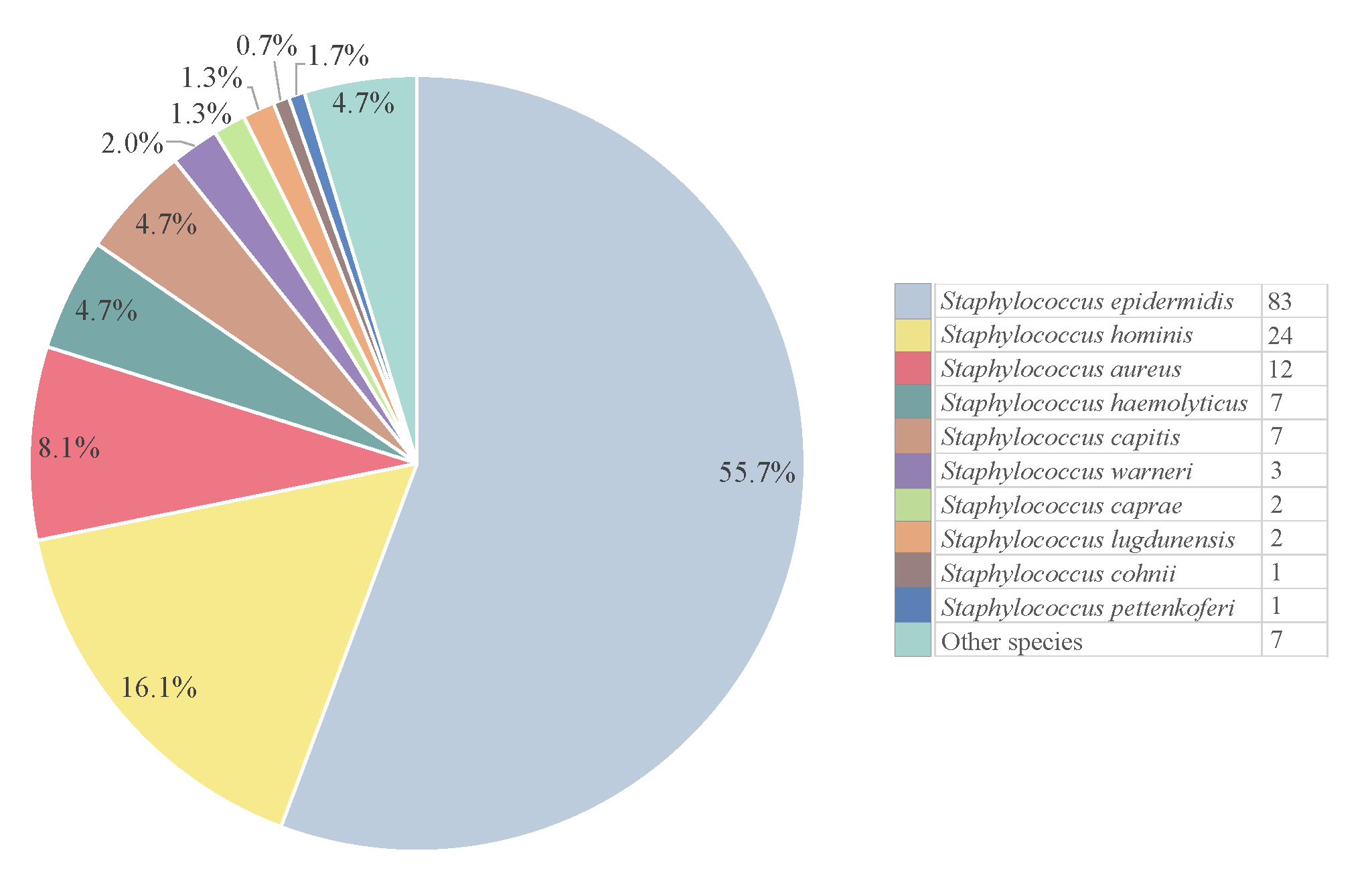


**Supplementary Figure 2.** Isolates profiles. The number and proportion of strains were showed in the table and graph, respectively.


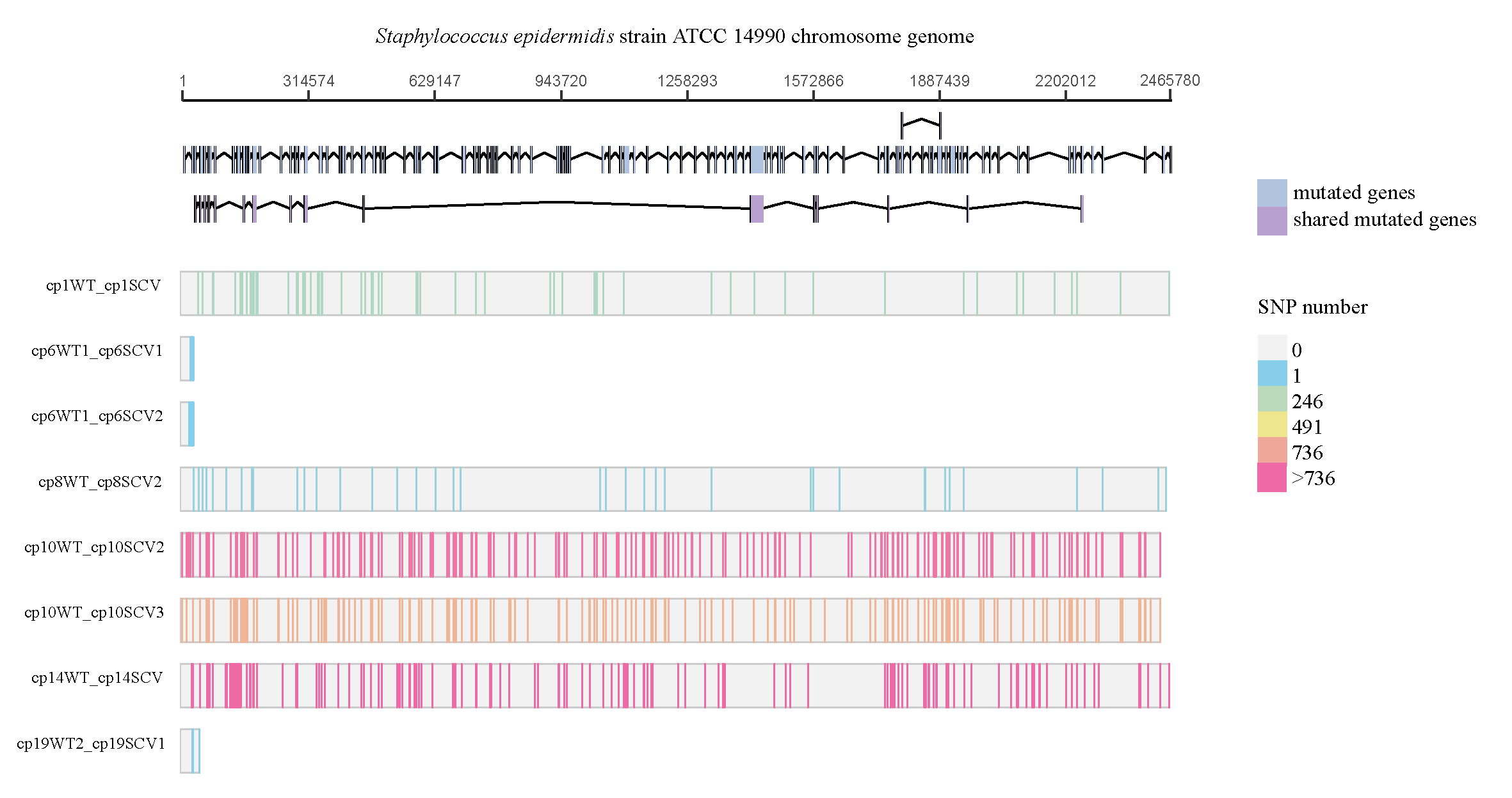


**Supplementary Figure 3.** Single nucleotide polymorphism (SNP) density map of 8 pairs of *S. epidermidis* WT and SCVs. *S. epidermidis* strain ATCC 14990 was used as reference. SNP number of each pair strains was displayed by the vertical stripes of different colors, including CDS of genes and intergenic regions. All mutated genes and the genes shared by two or more patients were highlighted by light blue and light purple, respectively.

## Supplementary Tables

**Supplementary Table 1.** Antimicrobial susceptibility testing results of staphylococcus strains

|  | Total  (n=142) | | | CoNS  (n=130) | | | *Staphylococcus aureus*  (n=12) | | |
| --- | --- | --- | --- | --- | --- | --- | --- | --- | --- |
| MRS (%) | 53.2 | | | 55.1 | | | 33.3 | | |
|  | **R%** | **S%** | **I%** | **R%** | **S%** | **I%** | **R%** | **S%** | **I%** |
| penicillin | 84.3 | 15.7 |  | 84.4 | 15.6 |  | 83.3 | 16.7 |  |
| oxacillin | 53.2 | 46.8 |  | 55.1 | 44.9 |  | 33.3 | 66.7 |  |
| ceftaroline |  |  |  |  |  |  |  | 100 |  |
| teicoplanin |  | 97.8 | 2.2 |  | 97.6 | 2.4 |  | 100 |  |
| vancomycin |  | 100 |  |  | 100 |  |  | 100 |  |
| daptomycin |  | 100 |  |  | 100 |  |  | 100 |  |
| erythromycin | 67.4 | 32.6 |  | 66.9 | 33.1 |  | 72.7 | 27.3 |  |
| gentamicin | 5.6 | 90.8 | 3.5 | 3.8 | 93.1 | 3.1 | 25 | 66.7 | 8.3 |
| clindamycin | 43.5 | 55.8 | 0.7 | 40.5 | 58.7 | 0.8 | 75 | 25 |  |
| linezolid |  | 100 |  |  | 100 |  |  | 100 |  |
| tigecycline |  | 100 |  |  | 100 |  |  | 100 |  |
| levofloxacin | 32.4 | 66.2 | 1.4 | 33.8 | 64.6 | 1.5 | 16.7 | 83.3 |  |
| moxifloxacin | 9.2 | 67.4 | 23.4 | 10.1 | 65.9 | 24 |  | 83.3 | 16.7 |
| rifampin | 4.9 | 95.1 |  | 5.4 | 94.6 |  |  | 100 |  |
| trimethoprim-sulfamethoxazole | 33.8 | 66.2 |  | 33.1 | 66.9 |  | 41.7 | 58.3 |  |

Abbreviations: CoNS, coagulase-negative staphylococci; MRS, methicillin-resistant staphylococcus; %R, % of isolates resistant; %S, % of isolates susceptible; %I, % of isolates intermediate. The duplicate susceptibility results from one patient’s different specimens were removed.

**Supplementary Table 2.** Antimicrobial susceptibility testing results of coagulase negative staphylococcus SCVs

|  | CoNS WT  (n=107) | | | CoNS SCVs  (n=23) | | |
| --- | --- | --- | --- | --- | --- | --- |
| MRS (%) | 57.1 | | | 45.5 | | |
|  | **R%** | **S%** | **I%** | **R%** | **S%** | **I%** |
| penicillin | 85.7 | 14.3 |  | 78.3 | 21.7 |  |
| oxacillin | 57.1 | 42.9 |  | 45.5 | 54.5 |  |
| teicoplanin |  | 98.1 | 1.9 |  | 95.7 | 4.3 |
| vancomycin |  | 100 |  |  | 100 |  |
| daptomycin |  | 100 |  |  | 100 |  |
| erythromycin | 73.8 | 26.2 |  | 34.8 | 65.2 |  |
| gentamicin | 4.7 | 92.5 | 2.8 |  | 95.7 | 4.3 |
| clindamycin | 45.6 | 54.4 |  | 17.4 | 78.3 | 4.3 |
| linezolid |  | 100 |  |  | 100 |  |
| tigecycline |  | 100 |  |  | 100 |  |
| levofloxacin | 33.6 | 64.5 | 1.9 | 34.8 | 65.2 |  |
| moxifloxacin | 10.4 | 67 | 22.6 | 8.7 | 60.9 | 30.4 |
| rifampin | 4.7 | 95.3 |  | 8.7 | 91.3 |  |
| trimethoprim-sulfamethoxazole | 33.6 | 66.4 |  | 30.4 | 69.6 |  |

Abbreviations: CoNS, coagulase-negative staphylococci; SCVs, small colony variants; WT, wild-type strains; MRS, methicillin-resistant staphylococcus; %R, % of isolates resistant; %S, % of isolates susceptible; %I, % of isolates intermediate. The duplicate susceptibility results from one patient’s different specimens were removed.
